# AlphaFold distillation for inverse protein design

**DOI:** 10.1038/s41598-025-00436-1

**Published:** 2025-07-01

**Authors:** Igor Melnyk, Aurélie Lozano, Payel Das, Vijil Chenthamarakshan

**Affiliations:** IBM Research, Yorktown Heights, NY 10598 USA

**Keywords:** Machine learning, Protein design

## Abstract

Inverse protein folding, the process of designing sequences that fold into a specific 3D structure, is crucial in bio-engineering and drug discovery. Traditional methods rely on experimentally resolved structures, but these cover only a small fraction of protein sequences. Forward folding models like AlphaFold offer a potential solution by accurately predicting structures from sequences. However, these models are too slow for integration into the optimization loop of inverse folding models during training. To address this, we propose using knowledge distillation on folding model confidence metrics, such as pTM or pLDDT scores, to create a faster and end-to-end differentiable distilled model. This model can then be used as a structure consistency regularizer in training the inverse folding model. Our technique is versatile and can be applied to other design tasks, such as sequence-based protein infilling. Experimental results show that our method outperforms non-regularized baselines, yielding up to 3% improvement in sequence recovery and up to 45% improvement in protein diversity while maintaining structural consistency in generated sequences.

## Introduction

Eight of the top ten best-selling drugs are engineered proteins, making inverse protein folding a crucial challenge in bio-engineering and drug discovery ^1^. Inverse protein folding involves designing amino acid sequences that fold into a specific 3D structure. Computationally, this task is known as computational protein design and has been traditionally addressed by optimizing amino acid sequences against a physics-based scoring function ^2^. Recently, deep generative models have been introduced to learn the mapping from protein structure to sequences^3–8^. While these models often use high amino acid recovery, TM score, and low perplexity as success criteria, they overlook the primary goal of designing novel and *diverse* sequences that fold into the desired structure and exhibit novel functions.

In parallel, recent advancements have also greatly enhanced protein representation learning^9,10^, structure prediction from sequences^11,12^, and conditional protein sequence generation^13,14^. While inverse protein folding has traditionally focused on sequences with resolved structures, which represent less than 0.1% of known protein sequences, a recent study improved performance by training on millions of AlphaFold-predicted structures^7^. Despite this success, large-scale training is computationally expensive. A more efficient method could be to use a pre-trained forward folding model to guide the training of the inverse folding model.

**Fig. 1:**
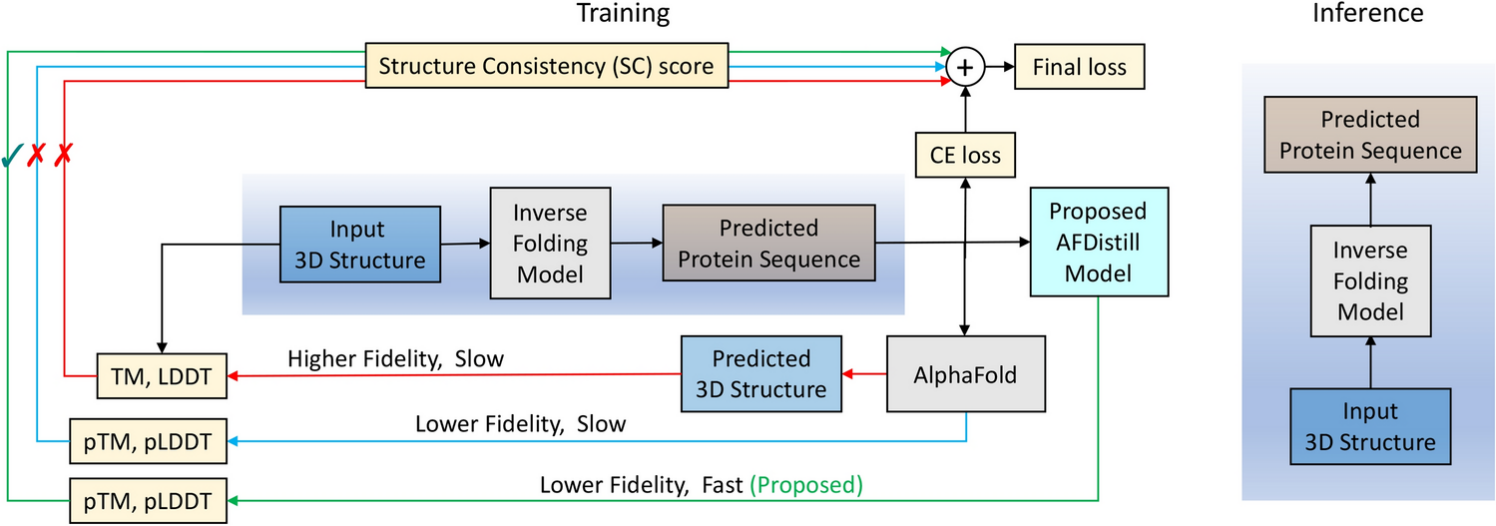
Overview of the proposed AFDistill system. AFDistill contrasts with traditional methods (red arrow) that use models like AlphaFold to predict protein structure, which is then compared to the actual structure. Such methods are slow due to model inference times (refer Fig. 2). An alternative (blue line) uses internal metrics from folding model without structure prediction but remains slow and less precise. Our solution, distills AlphaFold’s confidence metrics into a faster, differentiable model that offers accuracy akin to AlphaFold, allowing seamless integration into the training process (green arrow). The improved inverse folding model’s inference is shown on the right.

**Fig. 2:**
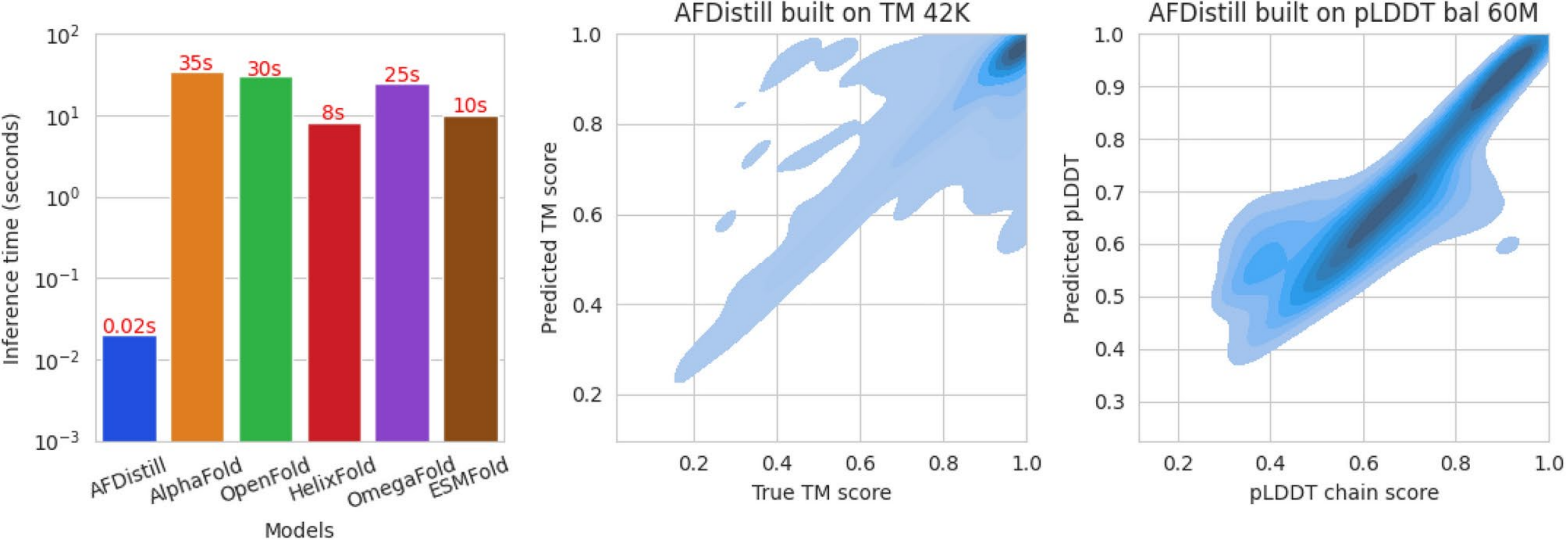
Inference times for protein sequences using our AFDistill model compared to alternatives are displayed on the left. AFDistill maintains fast inference for longer sequences: 0.028s for 1024-length and 0.035s for 2048-length. Timings for AlphaFold and OpenFold ^16^ do not include MSA search times, which can range from minutes to hours. Values for HelixFold ^17^, OmegaFold ^18^, and ESMFold ^19^ are from their publications. The center plot shows kernel density of true vs. AFDistill-predicted TM scores (Pearson’s correlation: 0.77), while the right displays a similar plot for pLDDT values (Pearson’s correlation: 0.76). Refer to Section "AlphaFold distill" for details.

In this work we construct a framework where the inverse folding model is trained using a loss objective that consists of a regular sequence reconstruction loss, augmented with an additional *structure consistency loss (SC)* (see Fig. 1 for system overview). One way of implementing this would be to use folding models, e.g., AlphaFold, to estimate the structure from a generated sequence, compare it with the ground truth and compute the TM score to regularize the training. However, a challenge in using Alphafold (or similar) directly is the computational cost of inference (see Fig. 2), and the need of ground truth reference structure. Internal confidence structure metrics from the folding model can be used instead. However, that approach is still slow for in-the-loop optimization. To address this, we make the following contributions.

**(i)** We propose to carry out knowledge distillation on AlphaFold and incorporate the resulting model, AFDistill (fixed), into the regularized training of the inverse folding model, which is referred to as structure consistency (SC) loss. The major *novelty* here is that AFDistill enables direct prediction of TM or LDDT scores of a given protein sequence bypassing the structure estimation or the access to ground truth structure. Primary practical benefits of our model include being fast, precise, and end-to-end differentiable. Employing SC loss during training for downstream tasks can be seen as integrating AlphaFold’s domain expertise into the model, thereby offering additional boost in its performance.

**(ii)** We perform extensive evaluations, demonstrating that our proposed system surpasses existing benchmarks in structure-guided protein sequence design by achieving lower perplexity, higher amino acid recovery, and maintaining proximity to the original protein structure. Additionally, our system enhances sequence diversity, a key objective in protein design. Due to a trade-off between sequence and structure recovery, our regularized model offers better sequence diversity while maintaining structural integrity. Importantly, our regularization technique is versatile, as evidenced by its successful application in sequence-based protein infilling, where we also observe performance improvement.

**(iii)** Lastly, our SC metric can either be used as regularization for inverse folding, infilling and other protein optimization tasks (e.g.,^15^) which would benefit from structural consistency estimation of the designed protein sequence, or as an affordable AlphaFold alternative that provides scoring of a given protein, reflecting its structural content.

## Results

### AlphaFold distill

Knowledge distillation^20^ transfers knowledge from a large complex model, in our case AlphaFold, to a smaller one, here this is the AFDistill model (see Fig.  9). Traditionally, the distillation would be done using soft labels, which are probabilities from AlphaFold model, and hard labels, the ground truth classes. However, in our case we do not use the probabilities as they are harder to collect or unavailable, but rather the model’s predictions (pTM/pLDDT) and the hard labels, TM/LDDT scores, computed based on AlphaFold’s predicted 3D structures. Note that TM/LDDT scores measure the similarity between the predicted and actual structure for a given protein sequence, while pTM and pLDDT are AlphaFold-estimated metrics, representing the model’s confidence in the estimated structure. We provide more details on the distilled scores in Section "Methods".

**Table 1:** Validation loss of AFDistill on datasets from Table 4 (For more details, see Supplemental Material, Tables 1 and 2).

Training data	Val CE loss	Training data	Val CE loss
TM 42K	**1.10**	LDDT 42K	3.39
TM augmented 86K	2.12	pLDDT 1M	3.24
pTM synthetic 1M	2.55	pLDDT balanced 1M	2.63
		pLDDT balanced 10M	2.43
		pLDDT balanced 60M	**2.40**

**Fig. 3:**
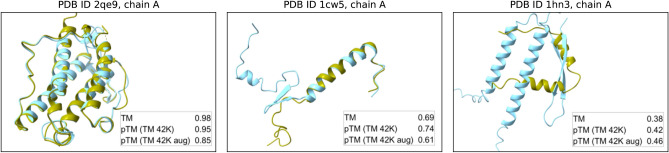
Examples of 3D protein structures from the dataset, corresponding to high, medium, and low actual TM scores (top row in legend), as well as AFDistill predictions, trained on TM 42K (middle row) and TM augmented 86K (bottom row).

**Fig. 4:**
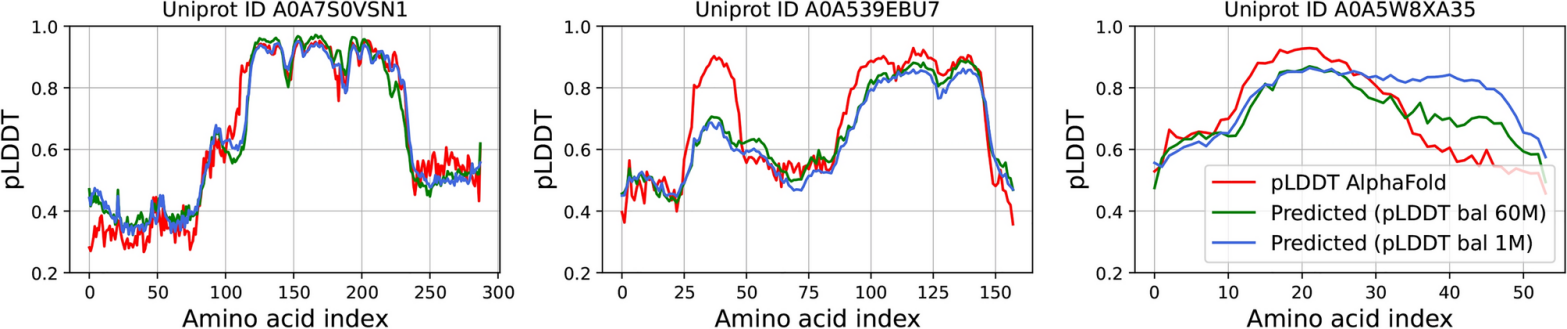
Dataset examples of the per-residue predictions for two AFDistill models (blue and green lines), build on pLDDT balanced 1M and 60M datasets, versus the AlphaFold predictions (red line).

In what follows, we evaluate the performance of AFDistill after training on a number of datasets (Section "Methods" discusses the details on data collection and curation). To address data imbalance, we used weighted sampling during minibatch generation and Focal loss^21^ instead of traditional cross-entropy loss. Table 1 shows results for (p)TM-based datasets. AFDistill trained on TM 42K performed the best, followed by augmented and synthetic data. For (p)LDDT-based datasets, increasing scale and data balancing improved validation performance.

In Fig. 2 we show kernel density plots of the true vs pTM scores and pLDDT values on the entire validation set. Majority of the density is concentrated along the diagonal, indicating that the predicted scores match well with the ground truth. The mismatches are grouped in off-diagonal regions, but these areas have low density, indicating that the predictions are still accurate. Moreover, since the data for TM 42K is skewed towards 1.0 (top density plot in Fig. 10), most of the data is clustered in upper left corner. On the other hand, for the dataset pLDDT bal 60M which is balanced (bottom panel in Fig. 10), the predictions and true values are spread more uniformly along the diagonal.

Finally, in Fig. 3 and 4, we show a few examples of the data samples together with the corresponding AFDistill predictions. Fig. 2 and Supplemental Material, Fig. 3 & 4 also show plots of SC (pTM or pLDDT) versus TM score, indicating that AFDistill is a viable tool for regularizing protein representation to enforce structural consistency or structural scoring of protein sequences, reflecting its overall composition and naturalness (in terms of plausible folded structure).

### Inverse protein folding design

In this section we demonstrate the benefit of applying AFDistill as a structure consistency (SC) score for solving the task of inverse protein folding. The overall framework is presented in Fig. 1 (following the green line in the diagram), where the traditional inverse folding model is regularized by our SC score.

**Fig. 5:**
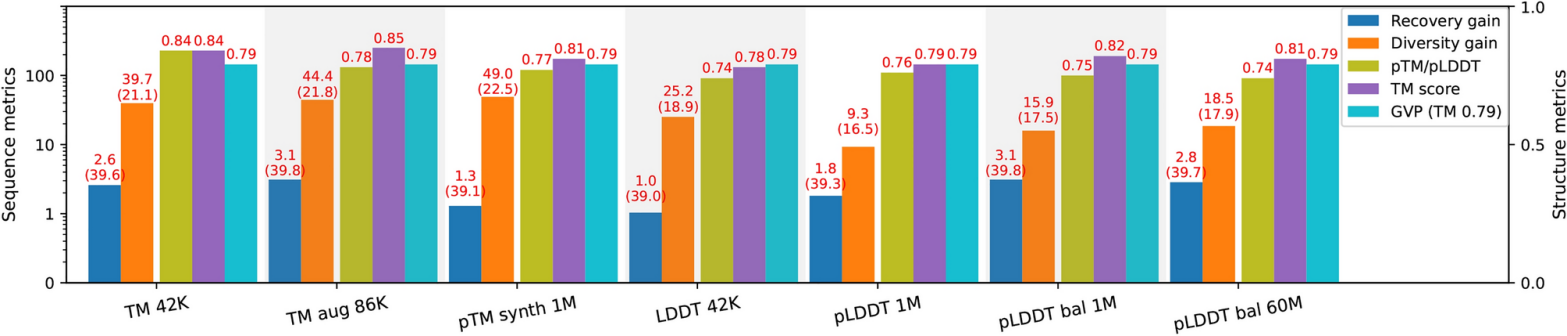
Evaluation results for GVP with SC regularization are shown with various AFDistill pretraining datasets on the x-axis. The left y-axis displays sequence metrics (recovery and diversity gains), and the right y-axis shows structure metrics (TM and SC scores). Blue and orange solid bars represent recovery and diversity gains over the vanilla GVP baseline (38.6 in recovery, 15.1 in diversity, and 0.79 in TM score). Olive and purple bars display predicted SC and test set TM scores, respectively, while dashed cyan bar shows the baseline GVP TM score. TM 42K and TM augmented 86K pretrained AFDistill models achieve the best overall performance, with high diversity and moderate improvement in sequence and structure recovery.

We use standard sequence evaluation metrics to measure prediction design quality. *Recovery* (0-100) is the normalized average of exact matches between predicted and ground truth sequences. *Diversity* (0-100) is the complement of the average recovery for pairwise comparisons in a set. We aim for high recovery and high diversity rates. *Perplexity* measures sequence likelihood, with lower values indicating better performance. For structure evaluation, we use *TM-score* and *structure consistency (SC)* score, which is AFDistill’s output for a given input.

We present experimental results for several recently proposed deep generative models for protein sequence design accounting for 3D structural constraints. For the inverse folding tasks we use CATH 4.2 dataset, curated by^22^. The training, validation, and test sets have 18204, 608, and 1120 structures, respectively. While for protein infilling we used SabDab^23^ dataset curated by^24^ and focus on infilling CDR-H3 loop. The dataset has 3896 training, 403 validation and 437 test sequences.

**GVP**

**Fig. 6:**
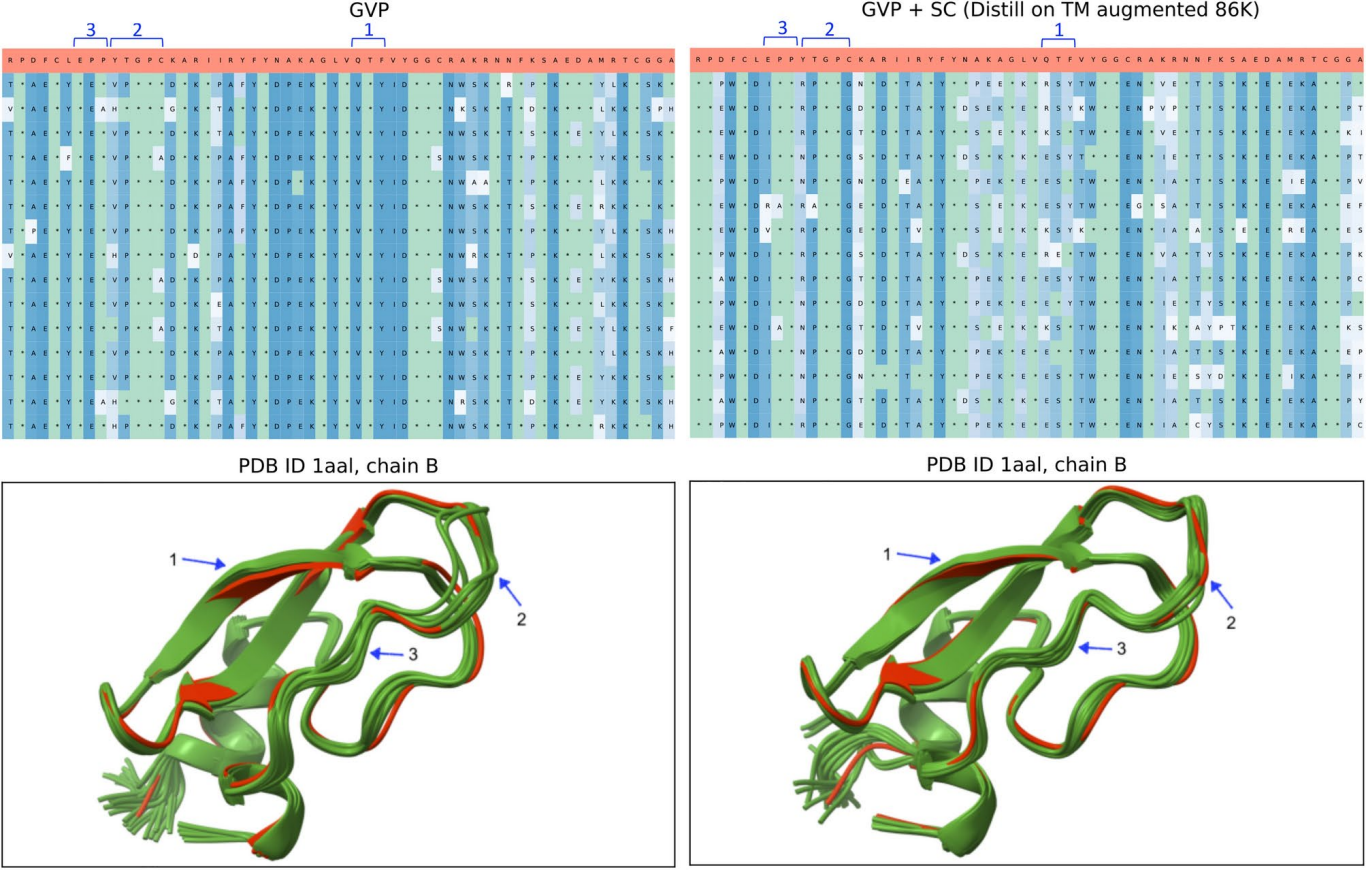
The comparison between baseline GVP (left) and SC regularized GVP (right) using AFDistill pre-trained on TM augmented 86K dataset shows 15 generated protein sequences from each model. Green cells with * indicate amino acid identity with ground truth (top red row), while blue cells represent novelty. The shade of blue indicates amino acid frequency in the column (darker = more frequent, lighter = rare). High recovery and diversity rates are seen with many green and light blue cells. Bottom plots display AlphaFold estimated structures (green) and ground truth (red). Recovery is 40.8 and diversity is 11.2 for GVP, while for GVP+SC, it is 42.8 and 22.6, respectively. SC-regularized GVP has accurate reconstructions with high sequence diversity, while GVP alone exhibits more inconsistencies, marked with arrows.

Geometric Vector Perceptron GNNs (GVP) ^3^ is the inverse folding model, that for a given target backbone structure, represented as a graph over the residues, replaces dense layers in a GNN by simpler layers, called GVP layers, directly leveraging both scalar and geometric features. This allows for the embedding of geometric information at nodes and edges without reducing such information to scalars that may not fully capture complex geometry. The results of augmenting GVP training with SC score regularization are shown in Fig. 5 (see also Supplemental Material, Table 9 for additional results).

Baseline GVP without regularization achieves 38.6 in recovery, 15.1 in diversity, and 0.79 in TM score on the test set. Employing SC regularization leads to consistent improvements in sequence recovery (1-3%) and significant diversity gain (up to 45%) while maintaining high TM scores. pTM-based SC scores show a better overall influence on performance compared to pLDDT-based ones. It’s important to note that AFDistill’s validation performance on distillation data doesn’t always reflect downstream application performance. For example, TM augmented 86K outperforms TM 42K, despite having slightly worse validation CE loss. This suggests that augmented models may enable more generalized sequence-structure learning and provide a greater performance boost for inverse folding models.

**Fig. 7:**
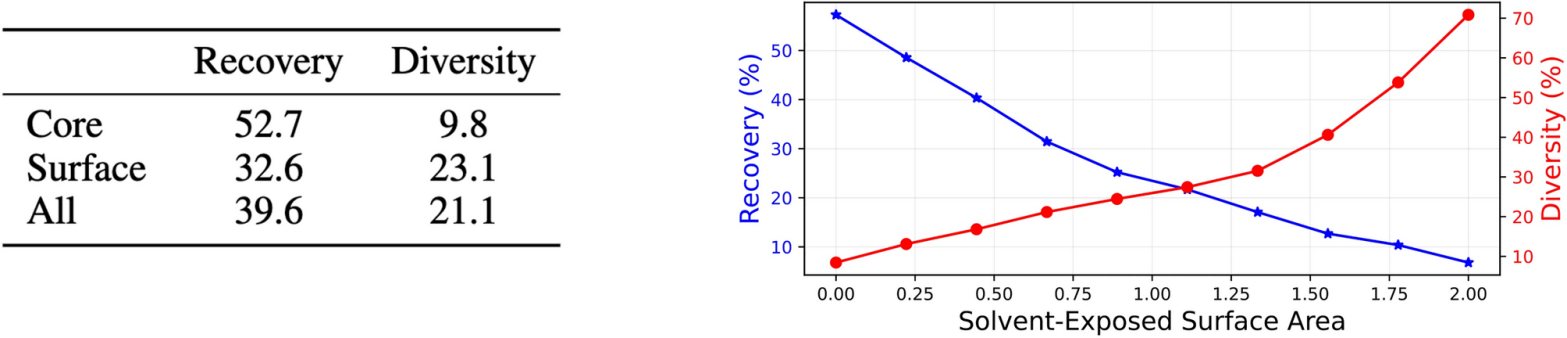
Distribution of recovery and diversity values across residues in generated structures based on the CATH 4.2 test set. Left Table shows the analsys with respect to core ($\ge$ 24 neighbors), surface ($\le$ 16 neighbors) and all residue categories. Right Figure shows similar analysis with respect to solvent-exposed surface area.

Fig. 6 further demonstrates the effect of recovery and diversity on protein sequences and AlphaFold-generated 3D structures for GVP and GVP+SC models. GVP+SC achieves higher diversity while retaining accurate structure. Recovery and diversity scores are 40.8 and 11.2 for GVP, and 42.8 and 22.6 for GVP+SC, respectively. The bottom plots display AlphaFold estimated structures (green) and ground truth (red). Despite its high sequence diversity, GVP+SC shows very accurate reconstructions (average TM score 0.95), while GVP exhibits more inconsistencies (TM score 0.92), marked with blue arrows.

**Table 2:** Evaluation results of ProteinMPNN trained with and without SC regularization (AFDistill trained on TM aug 86K dataset). The values in parenthesis show gain on test set of using SC-regularized training as compared to original training.

	Recovery		Diversity		Perplexity	
	ProteinMPNN	ProteinMPNN+SC	ProteinMPNN	ProteinMPNN+SC	ProteinMPNN	ProteinMPNN+SC
Backbone Noise 0.02	47.7	47.5 (-0.4%)	22.5	24.3 (+8.0%)	5.1	5.1 (+0.0%)
Backbone Noise 0.1	43.8	44.0 (+0.5%)	28.1	30.4 (+8.2%)	5.3	5.4 (+1.9%)
Backbone Noise 0.2	39.5	39.9 (+1.0%)	31.3	34.4 (+9.9%)	5.8	5.8 (+0.0%)
Backbone Noise 0.3	36.3	36.4 (+0.0%)	33.0	37.8 (+14.6%)	6.2	6.3 (+1.6%)

We also conducted a core/surface analysis similar to^7^ to examine recovery and diversity distribution across residues in generated structures. Residues were categorized by the density of neighboring $C_\alpha$ atoms within 10A (core: $\ge$ 24 neighbors; surface: $\le$ 16 neighbors). Using SC-regularized GVP results on TM 42K dataset, we computed recovery and diversity scores per class (Fig. 7). Core residues, being more constrained, better match ground truth with less diversity. Surface residues, being less constrained, exhibit lower recovery but higher diversity as the model has more freedom in selecting residues.

Additionally, we plot the solvent-exposed surface area^25^ versus recovery and diversity, computed per residue (see right panel in Fig. 7). As expected, the recovery plot shows negative correlation and diversity plot shows positive correlation as the surface area increases. Solvent-exposed surface area was calculated using GROMACS software suite^26^ with default parameters.

**Note on sequence diversity** In Section G of Supplemental Material we offer a set of experiments to shed some light on why SC regularization leads to improved sequence diversity. In particular in Supplemental Material, Fig.5 we show that the main source of diversity is in the limited guidance from AFDistill about the specific sequence to generate to match a given 3D structure, since it does not have access to the structural information, allowing many relevant sequences with high pTM/pLDDT to be considered as good candidates. AFDistill regularization during training injects candidate sequences which have high pTM/pLDDT scores, therefore likely matching the input structure better, thus ensuring high recovery rate. At the same time these sequences differ from the ground truth, thus promoting diversity (see Section G of Supplemental Material for more details).

**Fig. 8:**
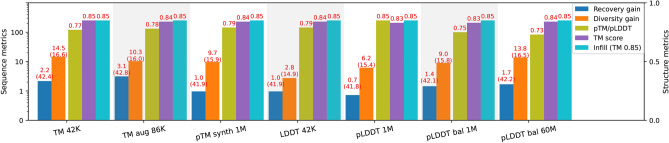
Evaluation results of Protein Infilling model trained with SC regularization. Baseline model achieves 41.5 in recovery, 14.5 in diversity and 0.85 in TM score on the test set. Similar as for the other applications, we see an improvement in the sequence recovery and even bigger gain in diversity. TM score shows that the resulting 3D structure remains close to the original, confirming the benefit of using SC score for training regularization.

Some studies, e.g.^27^, suggested that residue-level pLDDT scores are correlated with the likelihood of disorder. For instance, residues with low pLDDT scores are likely to be disordered and flexible, while high pLDDT scores would result in sequences that form more rigid structures than natural proteins. In our experiments we similarly observed that pTM-based SC regularization results in better performance as compared to pLDDT optimization, likely due to pTM being more global than pLDDT (see Figures 5 and 8). At the same time, SC loss in general does help maintain high actual TM to ground truth structure while delivering better sequence diversity (see Figure 7).

**ProteinMPNN** ProteinMPNN model^28^ is a recent protein design model, which is based on message passing neural network (MPNN) with specific modifications to improve amino acid sequences recovery of proteins given their backbone structures. The model incorporates structure features, edge updates, and an autoregressive approach for decoding the sequences. In Table 2 we compared the results of original unmodified training of ProteinMPNN to the SC-regulared training (AFDistill model trained on TM aug 86K dataset). We also varied ProteinMPNN internal parameter, which adds noise to the input backbone protein structure. As can be seen, SC regularization maintains high recovery and perplexity rates while improving the diversity of the generated protein sequences. Backbone noise, which is a part of ProteinMPNN model, can also be seen as a form of regularization, however while the increase in noise leads to improved sequence diversity it also leads to the decrease in amino acid recovery rate. SC regularization, on the other hand, promotes diverse generation and maintains high sequence recovery rates.

**PiFold** PiFold^29^ is another recent protein design model which introduces a new residue featurizer and stacked PiGNNs (protein inverse folding graph neural networks). The residue featurizer constructs residue features and introduces learnable virtual atoms to capture information that could be missed by real atoms. The PiGNN layer learns representations from multi-scale residue interactions by considering feature dependencies at the node, edge, and global levels. In Table 3 we present the results of original and SC-regularized PiFold (using different AFDistill models). We note that the original PiFold evaluation was based on using greedy decoding to generate a sequence. Following the standard practice (GVP, GraphTrans, ProteinMPNN, etc), we have included also the results based on sampling (using 100 samples per sequence) to match other works and compute sequence diversity score. The results show that SC regularization based on AFDistill trained on TM aug 86K results in a near-identical recovery rate compared to the original model, while notably enhancing sequence diversity. This indicates an improvement in PiFold’s performance by maintaining recovery rates while increasing the variety of generated sequences. Also observe a decrease in recovery rates for sampled generation as compared to greedy decoding across all the models.

**Table 3:** Experiments on PiFold comparing the performance metrics on the test set of CATH 4.2 for different model variants (original vs SC-regularized training based on different AFDistill models) using greedy and sampled decoding strategies. The values in parentheses represent the percentage change with respect to the original PiFold model.

	Original		TM 42K		TM aug 86K		TM synth 1M		LDDT 42K		pLDDT 1M		pLDDT bal 60M	
	Rec	Perp	Rec	Perp	Rec	Perp	Rec	Perp	Rec	Perp	Rec	Perp	Rec	Perp
Greedy	51.1	4.8	50.9	5.0	51.0	4.8	50.5	5.2	50.8	4.9	50.9	4.8	51.1	4.7
			(-0.4%)	(+4.0%)	(-0.2%)	(+0.0%)	(-1.2%)	(+8.3%)	(-0.6%)	(+2.1%)	(-0.4%)	(+0.0%)	(+0.0%)	(-2.1%)
	Rec	Div	Rec	Div	Rec	Div	Rec	Div	Rec	Div	Rec	Div	Rec	Div
Sampled	42.6	52.4	42.5	60.7	42.8	60.2	42.4	61.1	42.3	60.9	42.5	60.5	42.9	60.0
			(-0.2%)	(+15.8%)	(+0.5%)	(+14.9%)	(-0.5%)	(+16.6%)	(-0.7%)	(+16.2%)	(-0.2%)	(+15.5%)	(+0.7%)	(+14.5%)

In Section H in Supplemental Material we present additional experiments using ESM-IF^7^ and Graph Transformer ^5^ for the task of inverse protein flolding design.

**SC regularization for Protein Infilling**

Our proposed structure consistency regularization is quite general and not limited to the inverse folding task. Here we show its application on protein infilling task. Recall, that while the inverse folding task considers generating the entire protein sequence, conditioned on a given structure, infilling focuses on filling specific regions of a protein conditioned on a sequence/structure template. The complementarity-determining regions (CDRs) of an antibody protein are of particular interest as they determine the antigen binding affinity and specificity. We follow the framework of^24^ which formulates the problem as generation of the CDRs conditioned on a fixed framework region. We focus on CDR-H3 and use a baseline pretrained protein model ProtBERT ^30^ finetuned on the infilling dataset, and use ProtBERT+SC as an alternative (finetuned with SC regularization). The CDR-H3 is masked and the objective is to reconstruct it using the rest of the protein sequence as a template. The results are shown in Fig. 8 (see also Supplemental Material, Table 11 for additional results). Baseline model achieves 41.5 in recovery, 14.5 in diversity, and 0.80 in TM score on the test set. Similar as for the other applications, we see an improvement in the sequence recovery and even bigger gain in diversity, while using the AFDistill pretrained on TM 42K and TM augmented 86K, together with the pLDDT balanced datasets. TM score shows that the resulting 3D structure remains close to the original, confirming the benefit of using SC for training regularization.

It is worth noting that in general, modeling of the CDR-H3 loop is a challenging problem. AlphaFold is known to have lower prediction accuracy for these loops^31^ due to structure sensitivity even to a single amino acid change. However, as we show in our experiments, even under such settings, the in-filling design is able to benefit from SC regularization by promoting many diverse and still relevant loop candidates which are then selected by the in-filling optimization mechanism, resulting in the overall better performance as compared to the original in-filling approach.

## Conclusion

In this work we introduce AFDistill, a distillation model based on AlphaFold, which for a given protein sequence estimates its structural consistency (SC: pTM or pLDDT) score. AFDistill can then be used as a structure consistency regularizer in training an inverse folding model. We provide experimental results to showcase the efficiency and efficacy of the AFDistill model in high-quality protein sequence design, when used together with many of the current state of the art protein inverse folding models or large protein language model for sequence infilling. Our AFDistill model is fast and accurate enough so that it can be efficiently used for regularizing structural consistency in protein optimization tasks, maintaining sequence and structural integrity, while introducing diversity and variability in the generated proteins. In particular, we found that pTM-based structural consistency yields stronger performance in inverse folding tasks, likely because pTM is a global confidence measure capturing overall fold accuracy and structural design is a global problem, while pLDDT (a local, per-residue score) is beneficial in tasks focusing on local segments comprising a loop region (CDR loop infilling). Our experiments also underscored that a small high-quality training data can be more beneficial than a large noisy dataset. Indeed our TM 42K dataset, even though smaller, was curated to contain true PDB structures paired with AlphaFold predictions, providing high-fidelity training examples for matching exact TM or LDDT. In contrast, some of our larger synthetic or balanced sets included a mixture of artificially generated low-scoring sequences, which can help the model learn a broader distribution but may also introduce more noisy or uncertain examples.

## Methods

**Fig. 9:**
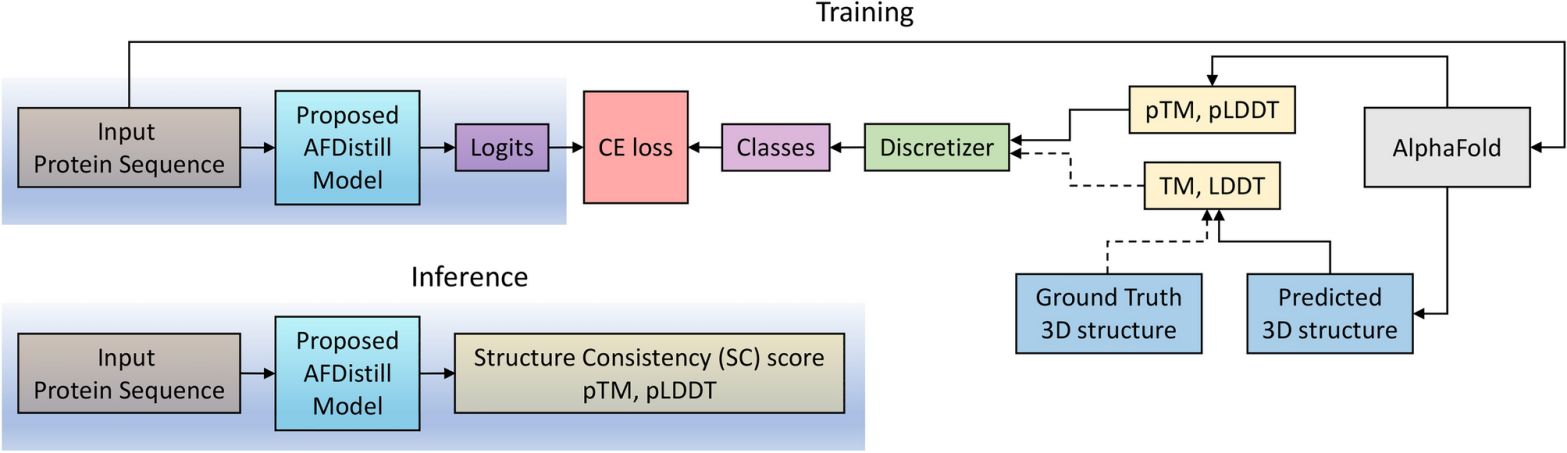
Distillation overview. Top diagram shows the training of AFDistill. The scores from AlphaFold’s confidence estimation are denoted as pTM and pLDDT, while the scores which are computed using ground truth and the AlphaFold’s predicted 3D structures are denoted as TM and LDDT. These values are then discretized and treated as class labels during cross-entropy (CE) training. Note that the training based on TM/LDTT is limited since the number of known ground truth structures is small. The bottom diagram shows the inference stage of AFDistill, where for each protein sequence it estimates pTM and pLDDT scores.

**Scores For Distillation** *TM-score*^32^ measures the mean distance between structurally aligned $C_\alpha$ atoms, scaled by a length-dependent distance parameter, while *LDDT*^33^ calculates the average of four fractions using distances between atom pairs based on four tolerance thresholds within a 15Å inclusion radius. Both metrics range from 0 to 1, with higher values indicating more similar structures. *pTM* and *pLDDT* are AlphaFold-predicted metrics for a given protein sequence, representing the model’s confidence in the estimated structure. *pLDDT* is a local per-residue score, while *pTM* is a global confidence metric for overall chain reconstruction. In this work, we interpret these metrics as indicators of sequence quality or validity for downstream applications (see Section "Inverse protein folding design").

**AFDistill Training Dataset**

**Table 4:** Statistics from January 2022 (left side) and July 2022 (right size) releases of the AlphaFold database. For the earlier release, we created multiple datasets for pTM and pLDDT estimation, while for the later, larger release we curated datasets only for pLDDT estimation.

Release 3 (January 2022)		Release 4 (July 2022)	
Name	Size	Name	Size
Original	907,578	Original	214,687,406
TM 42K	42,605	pLDDT balanced 1M	1,000,000
TM augmented 86K	86,811	pLDDT balanced 10M	10,000,000
pTM synthetic 1M	1,244,788	pLDDT balanced 60M	66,736,124
LDDT 42K	42,605		
pLDDT 1M	905,850		

Using Release 3 (January 2022) of AlphaFold Protein Structure Database^34^, we collected a set of 907,578 predicted structures. Each of these predicted structures contains 3D coordinates of all the residue atoms as well as the per-resiude pLDDT confidence scores.

To avoid data leakage to the downstream applications, we first filtered out the structures that have 40% sequence similarity or more to the validation and test splits of CATH 4.2 dataset (discussed in Section "Inverse protein folding design"). The specific similarity value was selected similar to earlier studies, e.g.,^22,35,36^. Then, using the remaining structures, we created our pLDDT 1M dataset (see Table 4), where each protein sequence is paired with the sequence of per-residue pLDDTs. Additionally, to reduce the computational complexity of AFDistill training, we limited the maximum protein length to 500 by randomly cropping a subsequence.

**Fig. 10:**
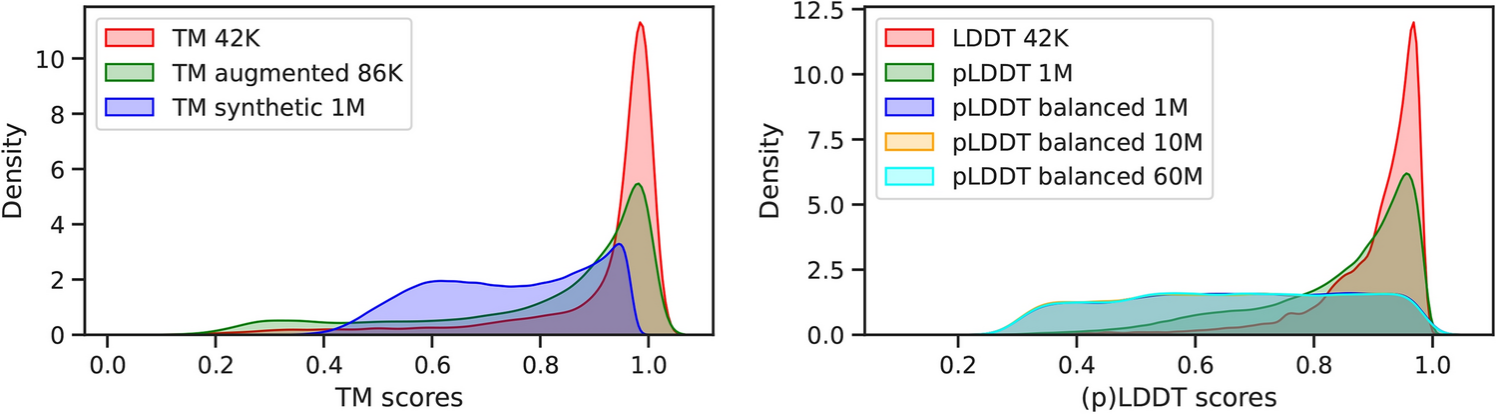
Distribution of the (p)TM/(p)LDDT scores in various datasets used in AFDistill training.

We created datasets based on true TM and LDDT values using predicted AlphaFold structures. Specifically, using the PDB-to-UniProt mapping, we selected a subset of samples with matching ground truth PDB sequences and 3D structures, resulting in 42,605 structures. We denote these datasets as TM 42K and LDDT 42K (see Table 4). Fig. 10 shows their score density distribution, which is skewed towards higher values. To address data imbalance, we curated two additional TM-based datasets. TM augmented 86K was obtained by augmenting TM 42K with a set of perturbed original protein sequences (permuted/replaced parts of protein sequence), estimating their structures with AlphaFold, computing corresponding TM-score, and keeping the low and medium range TM values. pTM synthetic 1M was obtained by generating random synthetic protein sequences and feeding them to AFDistill (pre-trained on TM 42K data), to generate additional data samples and collect lower-range pTM values. The distribution of the scores for these additional datasets is also shown in Fig. 10, where both TM augmented 86K and pTM synthetic 1M datasets are less skewed, covering lower (p)TM values better.

Using Release 4 (July 2022) with over 214M predicted structures, we observed a similar high skewness in pLDDT values. To mitigate this, we filtered out samples with upper-range mean-pLDDT values, resulting in a 60M sequences dataset, with additional 10M and 1M versions created. Their density is shown in Fig. 10.

In summary, AFDistill is trained to predict both the actual structural measures (TM, LDDT, computed using true and AlphaFold’s predicted structures) as well as AlphaFold’s estimated scores (pTM and pLDDT). In either case the estimated structural consistency (SC) score is well correlated with its target (refer to Fig.2) and can be used as an indicators of protein sequence quality or validity.

**AFDistill Model** AFDistill model is based on ProtBert^30^, a Transformer BERT model (420M parameters) pretrained on a large corpus of protein sequences using masked language modeling. For our task we modify ProtBert head by setting the vocabulary size to 50 (bins), corresponding to discretizing pTM/pLDDT in range (0,1). For pTM (scalar) the output corresponds to the first $\mathtt {\langle CLS \rangle }$ token of the output sequence, while for pLDDT (sequence) the predictions are made for each residue position. Note that AFDistill is not a full structure prediction model but a fast approximation that learns AlphaFold’s confidence metrics (pTM/pLDDT) for a given sequence. If AlphaFold struggles with low-homology proteins and produces unreliable pTM/pLDDT values, AFDistill will inherit this limitation since it learns from AlphaFold’s outputs. The accuracy of AFDistill is upper-bounded by AlphaFold’s confidence signals. However, the key advantage of AFDistill is that it serves as a lightweight, differentiable proxy for AlphaFold’s confidence, enabling faster training-time regularization for protein design tasks. This improves sequence diversity while maintaining structural consistency.

There are two main efficiency gains of using AFDistill: (1) Inference Speed: AFDistill’s forward pass takes on subseconds for sequences of length 1,000-2,000, whereas standard AF2 inference (and other folding approaches) typically requires tens of seconds to do the forward pass. AFDistill only outputs an estimate of pTM/pLDDT, while AF2 and other folding methods also generate the protein structure (see Figure 2) (2) Differentiable SC loss: AFDistill is a standard Transformer-based model, it can be included in the training loop, providing end-to-end backpropagation of a structural consistency gradient. AlphaFold was not designed for easy gradient-based integration as it is not differentiable.

**AFDistill as optimization loss** During training of the downstream applications, such as inverse protein design or antibody design, the candidate generated protein (as coming from the downstream method) is fed into AFDistill, for which it predicts pTM or pLDDT score, and is combined with the original downstream training objective (usually cross-entropy (CE) loss) results in

1 $$\begin{aligned} \mathcal {L} = \mathcal {L}_\text {CE} + \alpha \mathcal {L}_\text {SC}, \end{aligned}$$

where $\mathcal {L}_\text {CE} = \sum _{1}^N\mathcal {L}_\text {CE}(\textbf{s}_i, \hat{\textbf{s}}_i)$ is the CE loss, $\textbf{s}_i$ is the ground truth and $\hat{\textbf{s}}_i$ is the generated protein sequence, $\mathcal {L}_\text {SC} = \sum _{i=1}^N (1-SC(\hat{\textbf{s}}_i))$ is the structure consistency loss, *N* the number of training sequences, and $\alpha$ is the weighting scalar for the SC loss, in all of our experiments it is set to 1.

## Supplementary Information

Supplementary Information.

## Data Availability

The code along with data sources and post-processing scripts are available at https://github.com/IBM/AFDistill.
