# Supplementary material for: AlphaFold distillation for inverse protein design
Source: Sci Rep. 2025 Jul 1;15:21743. doi: 10.1038/s41598-025-00436-1 (PMC12216305; doi:10.1038/s41598-025-00436-1)
Supplement: Supplementary file 1 — Supplementary Information. [file 41598_2025_436_MOESM1_ESM.pdf]

Supplementary Material for Paper Submission
AlphaFold Distillation for Inverse Protein Folding

Table of Contents

Supplementary Material **1**

A Limitations of the Proposed Work **2**

B Background on Protein Design **2**

C AlphaFold Model Overview **2**

D AFDistill Training **3**

E AFDistill scatter plots of predictions **3**

F Architectural and Training Details **4**

F.1 AFDistill 4

F.2 Protein Design 5

G GVP Training Details **6**

G.1 Effect Of Using AFDistill Trained From Scratch 6

G.2 Effect of Structure Consistency (SC) Score On GVP Performance 6

H Additional Performance Comparisons of SC Regularization **8**

H.1 ESM-IF 8

H.2 Graph Transformer 9

H.3 Protein Infilling 9

I AFDistill Evaluation on Downstream Applications **10**

A LIMITATIONS OF THE PROPOSED WORK

Although our proposed AFDistill system is novel, efficient and showed promising results during evaluations, there are a number of limitations of the current approach:

- AFDistill dependency on the accuracy of the AlphaFold forward folding model: The quality of the distilled model is directly related to the accuracy of the original forward folding model, including the biases inherited from it.
- Limited coverage of protein sequence space: Despite the advances in AlphaFold forward folding models, they are still limited in their ability to accurately predict the structure of many protein sequences, including the TM score and pLDDT confidence metrics, that AFDistill relies on.
- Uncertainty in structural predictions: The confidence metrics (TM score and pLDDT) used in the distillation process are subject to uncertainty, which can lead to errors in the distilled model's predictions and ultimately impact the quality of the generated sequences in downstream applications.
- The need for a large amount of computational resources: The training process of AFDistill model requires significant computational resources. However, this might be mitigated by the amortization effect where the high upfront training cost in downstream applications pays in terms of cheap and fast inference through the model.

B BACKGROUND ON PROTEIN DESIGN

A protein is a linear chain of variable length made up of twenty amino acids, also called residues. These are denoted by 20 characters (A-Alanine, G-Glycine, I-Isoleucine, L-Leucine, P-Proline, V-Valine, F-Phenylalanine, W-Tryphtophan, Y-Tyrosine, D-Aspartic Acid, E-Glutamic Acid, R-Arginine, H-Histidine, K-Lysine, S-Serine, T-Threonine, C-Cystene, M-Methionine, N-Asparagine, Q-Glutamine). Each amino acid has the same core structure (backbone), consisting of alpha carbon atom C_α, connected to an amino group $NH2$, carboxyl group $COOH$ and hydrogen atom H. The backbone is identical in all amino acids, while the variable group, called side chain, which is also attached to alpha carbon C_α, is always different and determines the amino acid, including its chemical and mechanical properties. Amino acids are attached to each other by a covalent bond, known as peptide bond (carboxyl group $COOH$ of one amino acid and the amino group $NH2$ of the other amino acid combine, releasing water molecule $H2O$ and create a peptide bond). In this work, as is commonly done, we define protein 3D structure specified only by the C_α atoms of amino acids.

The protein inverse folding task is to draw a sequence from the true distribution of n-length sequences of amino acids $Y \in \{1, \dots, 20\}$, conditioned on a fixed protein structure, such that the designed protein folds into that structure. The protein structure can be represented as an attributed graph $G = (V, E)$ with node features $V = \{v_1, \dots, v_N\}$, describing each residue and edge features $E = \{e_{ij}\}$, capturing relationships between them. Thus, the final conditional distribution we are interested in modeling is: $P(Y|X) = p(y_i, \dots, y_n|X)$, which is known as computational protein design task.

Protein structures are intrinsically dynamic and each structure thus possess high designability, i.e. the total number of amino acid sequences that can fold to a target protein structure is high, without losing stability of the structure. The highly designable structures always enjoy beneficial properties such as higher thermodynamic stability, mutational stability, fast folding, functional robustness, etc. Therefore, we need to learn a "soft" function that can model this high designability associated with a protein structure, i.e. generating diverse sequences for a given protein structure.

C ALPHAFOLD MODEL OVERVIEW

A schematic overview of AlphaFold model is shown in Fig. 1, which it takes as input a protein sequence and produces as output, among others, the predicted 3D structure, as well as the confidence estimates of its prediction, pTM and pLDDT, which measure the estimated confidence of how well the predicted and ground truth structures match.

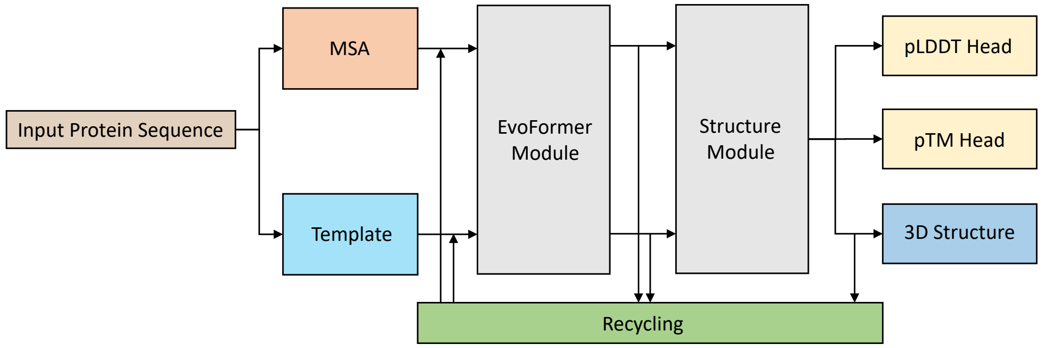

Figure 1: Overview of the inference stage in AlphaFold model. Given an input protein sequence, first the search is performed in genetic database to find similar sequences and construct multiple sequence alignments (MSA). Then a structure database search is done to find similar 3D structures and construct templates. The MSA and templates are fed into EvoFormer module, whose output is then sent to the Structure module, which is finally completed with the multiple output heads. The 3D structure head generates predicted 3D protein structure, while pLDDT and pTM heads estimate the confidence of the computed structure. Optionally, the generated structure together with the intermediate states are recycled and sent back to update/correct MSA and template representations for further processing and improvement.

D AFDistill Training

Tables 1, 2 show the validation performance of AFDistill trained on each of the (p)TM-based and (p)LDDT-based datasets, respectively. Table 3 shows results on (p)LDDT chain-based datasets. Note that (p)LDDT chain is the dataset, similar to (p)TM datasets, where for each sequence we associate a single scalar, in this case the average of all the per-reside (p)LDDT values.

E AFDistill scatter plots of predictions

In Fig. 2 we show scatter plots of the true vs pTM scores and pLDDT values on the entire validation set. We see a clear diagonal pattern in both plots, where the predicted and true values match. There are also some number of incorrect predictions (reflected along the off-diagonal), where we see that for the true scores in the upper range, the predicted scores are lower, indicating that AFDistill tends to underestimate them.

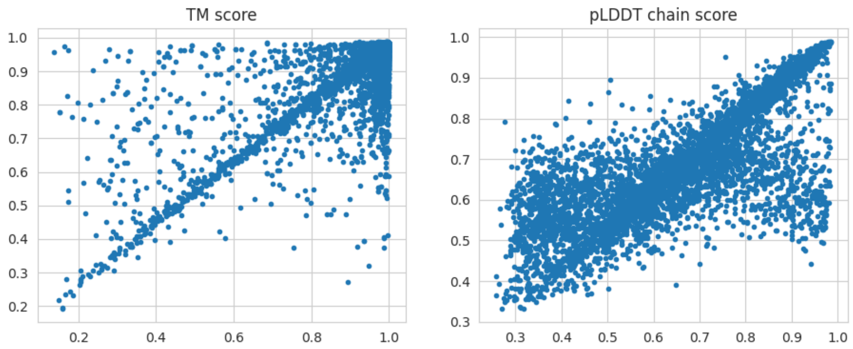

Figure 2: A scatter plot of the true and predicted TM score (left panel, data: TM 42K) and pLDDT (right panel, data: pLDDT bal 60M) for the data presented in main text in Fig.2.

Table 1: Validation CE loss for the AFDistill model trained on each of the (p)TM-based datasets. To address data imbalance during training, we employed weighted sampling for minibatch generation to so that the TM-scores cover their range (0,1) close to uniform distribution. Moreover, we also used Focal loss [1] in place of the standard cross-entropy (CE) loss (the evaluation is still done using CE loss across all the training setups). Based on the validation loss, we see that the AFDistill model trained on TM 42K dataset performed the best, followed by the dataset with augmentations, and the synthetic performed the worst. We also see that weighted sampling and focal loss do help in addressing the data imbalance problem, although for TM augmented 86K, the balanced augmentation seemed to help better and the best performance was for the case when no weighted sampling is applied and the traditional CE loss is used. As shown in Section 2.2 of the main article, the validation performance on the distillation data may not always indicate the performance on the downstream applications, where in particular we observed that the Distill model, trained on TM augmented 86K dataset, overall performed better than TM 42K, while having slightly worse validation CE loss.

Data	Training		Validation
	Weighted sampling	Focal loss (γ)	CE loss
TM 42K	–	–	1.33
	+	–	1.37
	+	1.0	1.16
	+	3.0	**1.10**
	+	10.0	1.29
TM augmented 86K	–	–	**2.12**
	+	1.0	2.15
	+	3.0	2.19
	+	10.0	2.25
pTM synthetic 1M	–	–	2.90
	+	1.0	2.75
	+	3.0	**2.55**
	+	10.0	3.20

Table 2: Validation CE loss for AFDistill trained on each of the (p)LDDT-based datasets. We see that weighted sampling coupled with Focal loss, performed the best.

Data	Training		Validation
	Weighted sampling	Focal loss (γ)	CE loss
LDDT 42K	-	-	3.47
	+	1.0	3.44
	+	3.0	3.42
	+	10.0	**3.39**
pLDDT 1M	-	-	3.27
	+	1.0	3.28
	+	3.0	**3.25**
	+	10.0	3.24

F Architectural and Training Details

F.1 AFDistill

Table 4 shows architectural details of AFDistill and ProtBert, while in Table 5 we present training details for AFDistill for two experimental setups. In all the experiments we used A100 GPUs. From the tables we can see that the AFDistill (420 M parameters) training takes approximately 24 hours on 1 GPU for TM 42K dataset, and 7 days on 8 GPUs for pLDDT balanced 60M dataset. Note

Table 3: Validation CE loss for the AFDistill model trained on each of the (p)LDDT chain-based datasets. (p)LDDT chain is the dataset, similar to (p)TM datasets, where for each sequence we associate a single scalar, in this case the average of all the per-reside (p)LDDT values. Similar as before, we see that the use of weighted sampling coupled with Focal loss helps in boosting the model performance. We also see that increasing the scale of data (which is already balanced) improves the performance even further.

Data	Training		Validation
	Weighted Sampling	Focal loss (γ)	CE loss
LDDT chain 42K	-	-	3.69
	+	1.0	3.57
	+	3.0	3.63
	+	10.0	**3.59**
pLDDT chain 1M	-	-	3.29
	+	1.0	3.36
	+	3.0	**3.30**
	+	10.0	3.31
pLDDT chain balanced 1M	–	–	2.45
pLDDT chain balanced 10M	–	–	2.24
pLDDT chain balanced 60M	–	–	**2.21**

that AFDistill training cost is amortized: the model is trained once and reused it many downstream applications. During training of the downstream applications, AFDistill model needs to be kept in memory to compute SC (structural consistency) score.

Table 4: Architectural details of AFDistill and ProtBert (which is used to initialize AFDistill training).

Model	Number of parameters	Number of layers	Hidden layer size	Number of heads	Vocab size	Pretraining Data	Reference
ProtBert	420M	30	1024	16	30 (20 amino acids + 10 aux tokens)	BFD100 (572 GB, 2B proteins)<br>Uniref100 (150 GB, 216M proteins)	[2]
AFDistill	420M	30	1024	16	50 (50 bins, TM/pLDDT (0,1))	TM 42K (20 MB, 42K sequences)<br>pLDDT balanced 60M (100 GB, 60M sequences)	–

Table 5: Training details for AFDistill for two experimental setups: small - using TM 42K dataset, and large - using AFDistill pLDDT balanced 60M.

Model	Learning rate	Batch size	Optimizer	GPUs	Training time
AFDistill TM 42K	$1e^{-6}$	10	Adam	1 × A100, 40GB	1 day
AFDistill pLDDT bal 60M	$1e^{-6}$	10	Adam	8 × A100, 40GB	7 days

F.2 PROTEIN DESIGN

Table 6 shows training details for GVP and ProteinMPNN models. Additionally, we note that for the original PiFold model it takes 60 epochs (6 hours on 1 GPU) to train the model, while for PiFold+SC it takes 60 epochs (8 hours on 1 GPU) to do the training. The increased training time is due to frequent validations (which involves sampling 100 samples per sequence for recovery and diversity computations). Note, that once the downstream application is trained, AFDistill is not used during inference.

Table 6: Training details for GVP and ProteinMPNN (original, as well as SC-regularized using our AFDistill model). Note that although AFDistill has 420M parameters, these are not part of the learnable model parameters, therefore are not counted towards the total.

Setup	Parameters	Learning rate	Batch size	Optimizer	GPUs	Training time
GVP / GVP+SC	1M	$1e^{-3}$	3000 res/batch	Adam	1 × A100, 40GB	1 day
ProteinMPNN / ProteinMPNN + SC	1.6M	varied	5000 res/batch	Adam	1 × A100, 40GB	2 days

G GVP Training Details

An example of GVP training progress regularized by the structure consistency (SC) score computed by the AFDistill model (pre-trained on various (p)TM-based datasets) is shown in Fig. 3. This figure shows that although SC score may be less accurate on the absolute scale, on the relative scale we can see it accurately detecting decays and improvements in the sequence quality as the GVP trains. Similarly, in Fig. 4 we show scatter plots of estimated pTM versus true TM scorefor GVP-generated protein sequences regularized by SC score.

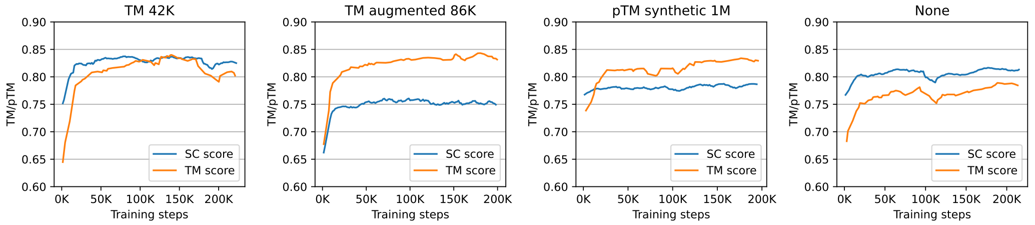

Figure 3: Example of the training progress (on CATH 4.2 dataset) of the GVP model regularized by the structure consistency (SC) score computed by the AFDistill model pre-trained on different datasets. Each plot shows the results for one of the Distill pre-training datasets, where the blue line represents the SC score computed by the AFDistill model (in this case generating pTM value), while the orange line shows the actual TM score computed between the ground truth structure and the AlphaFold's estimated 3D structures for a GVP-generated protein sequences. The last plot on the right shows the original, unregularized GVP training, where SC score was computed but never applied as part of the loss. It can be seen that SC correlates well with the TM score for TM 42K, while for others (TM augmented 86K and pTM synthetic 1M datasets) it tends to underestimate true TM score. Therefore, SC score may be less accurate on the absolute scale, while on the relative scale we can see that it can accurately detect decays and improvements in the sequence quality as the GVP trains. And the latter is of particular importance for SC to be a regularization loss during training, since it can clearly identify the ill-generated protein sequences early in the training (lower SC scores) and recognize well-defined sequences later during the training (higher SC scores).

G.1 Effect Of Using AFDistill Trained From Scratch

We also experimented with AFDistill models trained from scratch (as opposed to starting from pre-trained ProtBert), but observed worse performance. As an example, we trained AFDistill from scratch on TM42K dataset. The validation CE loss during distillation was 1.5 (versus 1.1 when using pre-trained ProtBert model). Moreover, training of AFDistill model from scratch takes longer (3 days vs 1 day). When regularizing GVP with AFDistill from scratch, we get similar recovery rate (39.4 vs 39.6) but lower sequence diversity (15.9 vs 21.1), which confirms the benefit of common practice of fine-tuning the pretrained models as opposed to starting from random models weights.

G.2 Effect of Structure Consistency (SC) Score On GVP Performance

For protein design (e.g., using GVP as a base model) the objective is CE + SC (cross-entropy + AFDistill structure consistency score). In Fig. 5 we present the effect of SC magnitude on the GVP performance on the test set of CATH dataset. As can be seen, when only the CE term is present (the blue left most bar in both panels, representing the original GVP), the model is encouraged to

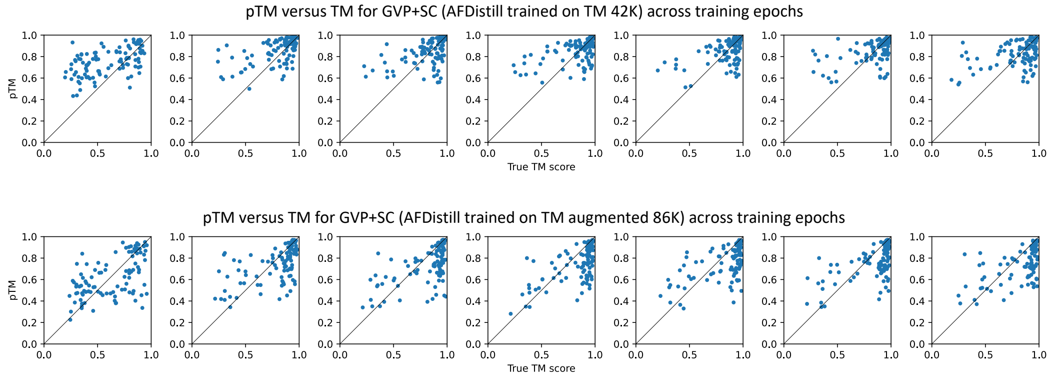

Figure 4: Estimated pTM versus true TM score (based on AlphaFold structure prediction) for GVP-generated protein sequences regularized by SC score. The top row shows results for SC computed by AFDistill model trained on TM 42K, while the bottom row is for AFDistill trained on TM augmented 86K. The columns in each row correspond to the progress as GVP trains. Note that the top row corresponds to the first left plot in Fig. 3, while the bottom row corresponds to the second plot in Fig. 3. It can be observed that in the earlier stages of GVP training, the generated protein sequences are of poor quality, reflected in pTM and TM scores that are spread across the (0,1) range. On the other hand, as the training progresses, the generated sequences are getting better and the pTM/TM score is concentrated more in the upper range. Another observation is that for AFDistill trained on TM 42K dataset, the predicted and true TM score are better aligned across the diagonal (compare with orange and blue lines on the left plot in Fig. 3), while for AFDistill trained on TM augmented 86K dataset, pTM tends to underestimate true TM score. These plots show that AFDistill is viable sequence scoring tool, which fairly accurately measures the structural consistency of the generated protein sequences. Combined with the fact that it is fast and end-to-end differentiable, shows its potential for many of the protein optimization problems.

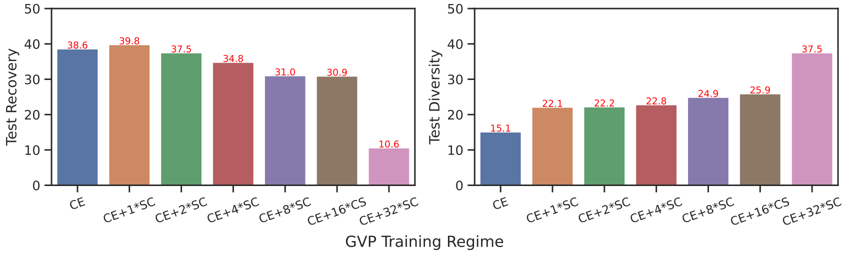

Figure 5: The effect of Structure Consistency (SC) loss on the performance of GVP. Left panel shows the amino acid recovery rate and the right panel shows the diveristy rate on the test set of CATH dataset. The horizontal y-axis shows the different choices of objective function during training: CE is the cross-entropy loss, SC is the Structure Consistency score computed by AFDistill.

recover the specific ground truth protein sequence for a given 3D structure, and this promotes model accuracy, and high amino acid recovery rate, while also resulting in low diversity. On the other hand, when only the SC term is present (the pink right most bar, reprenting CE+32*SC, i.e., when SC completely dominates and CE can be ignored), this results in poor and degenerated protein sequences. This is expected, since AFDistill alone cannot guide GVP which sequence it should generate to match the given input 3D structure. Recall, that AFDistill has no information about the structure, and since many of the relevant protein sequences can have high pTM/pLDDT, all of them could be good candidates, and this promotes high diversity and low recovery. Consequently, when both CE and SC terms are present and when appropriate balance between them is found (in our case it is CE+SC, corresponding to the orange bar in both panels), we get a full benefit, i.e., the accurate recovery and high diversity of the generated protein sequences.

	Model	Recovery	Recovery Change	Diversity	Diversity Change
1	GVP-GNN (1M) [3]	40.2	–	NA	–
2	GVP-GNN (1M) [4]	42.2	–	NA	–
3	GVP-GNN (1M) + AlphaFold2 data [4]	38.6	-3.6 (-8.5%)	NA	–
4	GVP-GNN (1M) (our experiment)	38.6	–	15.1	–
5	GVP-GNN (1M) + SC (our experiment)	39.6	+1.0 (+2.6%)	21.1	+6.0(+39.7%)
6	GVP-GNN-large (21M) [4]	39.2	–	NA	–
7	GVP-GNN-large (21M) (our experiment)	39.0	–	16.7	–
8	GVP-GNN-large (21M) + SC (our experiment)	40.1	+1.1(+2.8%)	19.3	+2.6(+15.6%)

Table 7: Comparison of amino acid recovery rate of protein sequences generated by GVP on the test split of CATH dataset. First row is the original result from GVP authors, rows 2 and 3 show the results from ESM authors, and row 4 shows the result from our experiments. A small difference between the values in first, second and forth rows can be attributed to some discrepancies in experimental settings as well as model initialization. We can see that a simple data augmentation baseline results in 3.6 (or 8.5%) drop of recovery relative to the unaugmented GVP (1M). On the other hand, the use of SC regularization leads to 1.0 (or 2.6%) gain in recovery, signaling the benefit of the proposed distillation approach. For the GVP-GNN-large (21M) model, shown in rows 6, 7 and 8 we were able to recover results closer to the published ones (39.0 vs their 39.2). And when SC is applied, we again see a boost in recovery (40.1 vs 39.0), and diversity (19.3 vs 16.7).

H Additional Performance Comparisons of SC Regularization

H.1 ESM-IF

In this Section we compare GVP-GNN (1M and 21M) performance under different training scenarios (CATH only and CATH + AlphaFold2 data) and present the results in Tables 7 and 8. The first row in Table 7 is the recovery rate of the original GVP-GNN (1M) model as reported in [3]. The following two rows (2 and 3) are the results presented in the work of [4] (ESM-IF). Their evaluation showed that the vanilla GVP achieved a slightly higher recovery rate of 42.2. GVP+AlphaFold2 represents the GVP trained on augmented dataset (CATH + AlphaFold2-generated structure/sequence pairs). Interestingly, this simple data augmentation baseline showed worse performance as compared to the original GVP, and the authors had to significantly increase GVP capacity (from 1M to 21M) to get any benefit from the data augmentation. Moreover, note that such a data augmentation idea can also serve as the baseline for our approach of AFDistill regularization, since AFDistill was trained on AlphaFold2-generated data and it can be thought of as a compressed representation of that data.

The rows (4 and 5) show our evaluation results of the vanilla GVP-GNN (1M), achieving slightly lower base recovery rate of 38.6, while this same GVP but trained with AFDistill regularization achieves a boost in recovery (39.6) and significant increase in the sequence diversity (+39.7% as we showed in Fig. 5 of the main article). On GVP-GNN-large (21M) model we were able to recover results closer to the published ones (39.0 vs their 39.2). And when SC is applied we again see a boost in recovery (40.1 vs 39.0), and diversity (19.3 vs 16.7).

Finally, in Table 8 we followed the setup of [4] and trained GVP-GNN-large (21M) on large dataset of CATH+AlphaFold2 (12M sequences) and evaluated on CATH test set. The second row in the table shows our that our experiment recovered results similar to the ones reported in [4], while in third row we present the SC-regularized training using our AFDistill model. Clearly, the sequence recovery was improved and even more so the diversity of the generated sequences went up from 13.8 to 18.5.

Therefore, comparing data augmentation and model distillation for the task of protein design, we see that for the GVP models (1M and 21M), AFDistill offers a clear advantage, providing a modest boost in recovery, while significantly increasing diversity of the generated sequences. This improvement

	Model	Recovery	Recovery Change	Diversity	Diversity Change
1	GVP-GNN-large (21M) [4]	50.8	–	NA	–
2	GVP-GNN-large (21M) (our experiment)	50.5	–	13.8	–
3	GVP-GNN-large (21M) + SC (our experiment)	50.9	+0.4(+0.8%)	18.5	+4.7(+34.0%)

Table 8: Comparison of amino acid recovery rate of protein sequences generated by GVP-GNN-large (21M) on the test split of CATH dataset, while trained on CATH + AlphaFold2 dataset (12M sequences). As observed before on experiment in GVP-GNN-large (21M) + SC when trained on CATH only, here when SC is applied, we see a minor boost in recovery (40.9 vs 50.5), and more significant increase in protein sequence diversity (13.8 vs 18.5).

after applying SC regularization occurs because the baseline techniques, which rely on CE in training, primarily emphasize accurate sequence recovery, neglecting other protein sequences that can achieve the desired structure. SC regularization encourages the consideration of many relevant and diverse protein sequences with high pTM/pLDDT scores as strong candidate sequences. This results in a moderate improvement in recovery and a significantly larger boost in diversity. Moreover, the distillation overhead is amortized, as we train AFDistill once and use it in many downstream applications. The data augmentation would require additional computational cost in every downstream application.

H.2 Graph Transformer

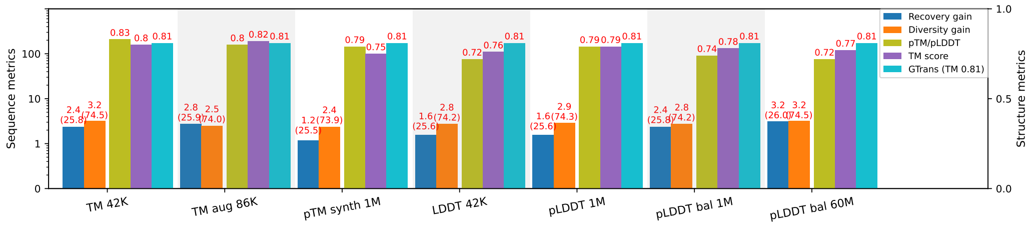

Figure 6: Evaluation results of Graph Transformer model trained with SC score regularization. Baseline model with no regularization achieves 25.2 in recovery, 72.2 in diversity and 0.81 in TM score on the test set.

We evaluated the effect of SC score on Graph Transformer [5], another inverse folding model, which seeks to improve standard GNNs to represent the protein 3D structure. Graph Transformer applies a permutation-invariant transformer module after GNN module to better represent the long-range pair-wise interactions between the graph nodes. The results of augmenting Graph Transformer training with SC score regularization are shown in Fig. 6 (see also Supplemental Material, Table 10 for additional results). Baseline model with no regularization has 25.2 in recovery, 72.2 in diversity and 0.81 in TM score on the test set. As compared to GVP (Fig. 5 in main), we can see that for this model, the recovery and diversity gains upon SC regularization are smaller. We also see that TM score of regularized model (TM 42K and TM augmented 86K pretraining) is slightly higher as compared to pLDDT-based models.

H.3 Protein Infilling

Our proposed structure consistency regularization is quite general and not limited to the inverse folding task. Here we show its application on protein infilling task. Recall, that while the inverse folding task considers generating the entire protein sequence, conditioned on a given structure, infilling focuses on filling specific regions of a protein conditioned on a sequence/structure template. The complementarity-determining regions (CDRs) of an antibody protein are of particular interest as they determine the antigen binding affinity and specificity. We follow the framework of [6] which

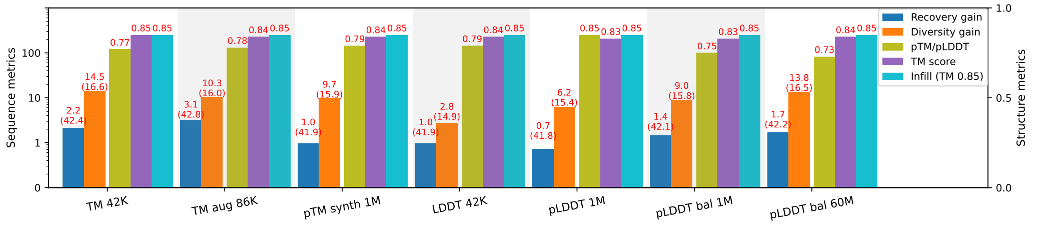

Figure 7: Evaluation results of Protein Infilling model trained with SC regularization. Baseline model achieves 41.5 in recovery, 14.5 in diversity and 0.85 in TM score on the test set. Similar as for the other applications, we see an improvement in the sequence recovery and even bigger gain in diversity. TM score shows that the resulting 3D structure remains close to the original, confirming the benefit of using SC score for training regularization.

formulates the problem as generation of the CDRs conditioned on a fixed framework region. We focus on CDR-H3 and use a baseline pretrained protein model ProtBERT [7] finetuned on the infilling dataset, and use ProtBERT+SC as an alternative (finetuned with SC regularization). The CDR-H3 is masked and the objective is to reconstruct it using the rest of the protein sequence as a template. The results are shown in Fig. 7 (see also Supplemental Material, Table 11 for additional results). Baseline model achieves 41.5 in recovery, 14.5 in diversity, and 0.80 in TM score on the test set. Similar as for the other applications, we see an improvement in the sequence recovery and even bigger gain in diversity, while using the AFDistill pretrained on TM 42K and TM augmented 86K, together with the pLDDT balanced datasets. TM score shows that the resulting 3D structure remains close to the original, confirming the benefit of using SC for training regularization.

I AFDistill Evaluation on Downstream Applications

Finally, in Tables 9 10, and 11 we show detailed results for GVP and Graph Transformer inverse folding task as well as protein infilling task. The table combines all the choices for AFDistill pretraining, showing their validation accuracy, and presents the corresponding performance on the downstream application without (top row in each table) and with SC regularization (all the following rows).

Distill model				GVP				
Data	Training: Weighted sampling	Training: Focal loss	Validation: CE loss	Recovery	Diversity	Perplexity	pTM/ pLDDT	TM score
–	–	–	–	38.6	15.1	6.1	0.80	0.79
TM 42K	+	–	1.37	36.8	22.2	6.3	0.78	
	+	1.0	1.16	37.6	21.1	6.0	0.87	
	+	3.0	1.10	**39.6**	21.1	5.9	0.84	0.84
	+	10.0	1.29	37.9	18.4	6.0	0.80	
TM augmented 86K	–	–	2.12	38.3	22.2	5.9	0.73	
	+	1.0	2.15	**39.8**	22.1	5.8	0.78	0.85
	+	3.0	2.19	37.8	19.8	6.1	0.73	
	+	10.0	2.25	38.5	21.2	5.9	0.72	
TM synthetic 1M	–	–	2.90	38.8	21.4	5.8	0.73	
	+	1.0	2.55	**39.1**	22.5	5.9	0.77	0.81
	+	3.0	2.75	39.0	21.9	5.8	0.74	
	+	10.0	3.20	39.0	22.0	5.9	0.69	
LDDT 42K	–	–	3.47	**39.0**	18.9	5.8	0.74	0.78
	+	1.0	3.44	38.7	22.5	5.8	0.73	
	+	3.0	3.42	38.9	21.2	5.8	0.73	
	+	10.0	3.39	38.5	22.3	5.9	0.72	
PLDDT 1M	–	–	3.27	**39.3**	16.5	5.9	0.76	0.79
	+	1.0	3.28	38.8	15.5	5.9	0.72	
	+	3.0	3.25	38.9	18.2	5.8	0.78	
	+	10.0	3.24	38.4	16.2	6.0	0.73	
LDDT chain 42K	–	–	3.69	38.8	20.0	5.8	0.74	
	+	1.0	3.57	**39.3**	16.3	5.8	0.79	0.78
	+	3.0	3.63	38.9	15.9	5.9	0.72	
	+	10.0	3.59	37.9	23.2	6.0	0.73	
pLDDT chain 1M	–	–	3.29	39.4	17.4	5.8	0.78	
	+	1.0	3.36	38.7	16.3	5.8	0.76	
	+	3.0	3.30	**39.6**	18.3	5.7	0.79	0.77
	+	10.0	3.31	38.2	20.1	6.0	0.76	
pLDDT balanced 1M	–	–	2.63	39.1	17.1	5.8	0.75	0.82
pLDDT balanced 10M	–	–	2.43	39.3	17.7	5.9	0.73	
pLDDT balanced 60M	–	–	2.40	**39.8**	17.5	5.9	0.74	0.81
pLDDT chain balanced 1M	–	–	2.45	38.6	16.6	5.9	0.73	
pLDDT chain balanced 10M	–	–	2.24	39.1	17.8	5.8	0.73	
pLDDT chain balanced 60M	–	–	2.21	**39.7**	17.9	5.9	0.74	0.82

Table 9: Evaluation results of GVP inverse folding task, trained without (top row) and with SC regularization (all other rows). The table combines all the choices for AFDistill pretraining and showing their validation accuracy, as well as the corresponding performance on the downstream application. We select the best performance for each experiment based on the highest recovery rate (marked in bold).

Distill model				Graph Transformer				
Data	Training		Validation	Recovery	Diversity	Perplexity	pTM/ pLDDT	TM score
	Weighted sampling	Focal loss	CE loss					
–	–	–	–	25.2	72.2	7.2	0.80	0.81
	+	–	1.37	24.1	74.4	7.4	0.81	
TM	+	1.0	1.16	25.2	73.2	7.2	0.86	
42K	+	3.0	1.10	**25.8**	74.5	7.2	0.83	0.80
	+	10.0	1.29	24.9	73.9	7.3	0.81	
	–	–	2.12	25.0	73.3	7.1	0.78	
TM augmented	+	1.0	2.15	**25.9**	74.0	7.1	0.80	0.82
86K	+	3.0	2.19	24.9	73.4	7.3	0.76	
	+	10.0	2.25	24.8	73.4	7.2	0.79	
	–	–	2.90	25.3	73.2	7.1	0.72	
TM synthetic	+	1.0	2.55	**25.5**	73.9	7.2	0.79	0.75
1M	+	3.0	2.75	25.2	73.5	7.2	0.77	
	+	10.0	3.20	24.9	74.2	7.2	0.76	
	–	–	3.47	25.4	73.2	7.1	0.75	
LDDT	+	1.0	3.44	**25.7**	74.2	7.1	0.72	0.76
42K	+	3.0	3.42	25.5	74.4	7.2	0.73	
	+	10.0	3.39	25.3	22.3	7.2	0.72	
	–	–	3.27	25.6	73.4	7.1	0.79	
pLDDT	+	1.0	3.28	25.4	74.1	7.2	0.78	
1M	+	3.0	3.25	**25.6**	74.3	7.1	0.79	0.79
	+	10.0	3.24	25.4	74.0	7.1	0.77	
	–	–	3.69	25.3	74.1	7.2	0.76	
LDDT chain	+	1.0	3.57	**25.8**	74.3	7.1	0.75	0.80
42K	+	3.0	3.63	25.5	74.2	7.1	0.77	
	+	10.0	3.59	25.6	74.1	7.2	0.76	
	–	–	3.29	25.3	74.3	7.1	0.78	
pLDDT chain	+	1.0	3.36	25.2	74.1	7.1	0.76	
1M	+	3.0	3.30	**25.6**	74.4	7.2	0.79	0.81
	+	10.0	3.31	25.3	74.3	7.1	0.77	
pLDDT balanced 1M	–	–	2.63	25.8	74.2	7.1	0.70	
pLDDT balanced 10M	–	–	2.43	25.7	74.5	7.1	0.73	
pLDDT balanced 60M	–	–	2.40	**26.0**	74.2	7.2	0.74	0.78
pLDDT chain balanced 1M	–	–	2.45	25.7	74.3	7.1	0.72	
pLDDT chain balanced 10M	–	–	2.24	25.9	74.4	7.1	0.74	
pLDDT chain balanced 60M	–	–	2.21	**25.9**	74.5	7.2	0.72	0.77

Table 10: Evaluation results of Graph Transformer inverse folding task, trained without (top row) and with SC regularization (all other rows). The table combines all the choices for AFDistill pretraining and showing their validation accuracy, as well as the corresponding performance on the downstream application. We select the best performance for each experiment based on the highest recovery rate (marked in bold).

Distill model				CDR Infill				
Data	Training		Validation	Recovery	Diversity	Perplexity	pTM/ pLDDT	TM score
	Weighted sampling	Focal loss	CE loss					
–	–	–	–	41.5	14.5	6.8	0.80	0.85
TM 42K	+	–	1.37	41.9	15.7	6.5	0.81	
	+	1.0	1.16	**42.4**	16.6	6.3	0.77	0.85
	+	3.0	1.10	41.7	14.6	6.7	0.78	
	+	10.0	1.29	40.8	14.4	6.6	0.79	
TM augmented 86K	–	–	2.12	**42.8**	15.5	6.5	0.78	0.84
	+	1.0	2.15	41.6	14.8	6.6	0.74	
	+	3.0	2.19	41.3	14.6	6.7	0.76	
	+	10.0	2.25	40.9	15.4	6.8	0.79	
TM synthetic 1M	–	–	2.90	41.8	16.0	6.6	0.79	
	+	1.0	2.55	**41.9**	15.9	6.7	0.79	0.84
	+	3.0	2.75	41.3	16.1	6.6	0.77	
	+	10.0	3.20	40.9	16.2	6.7	0.78	
LDDT 42K	–	–	3.47	41.3	15.1	6.5	0.83	
	+	1.0	3.44	40.3	15.5	6.7	0.84	
	+	3.0	3.42	40.8	14.4	6.8	0.81	
	+	10.0	3.39	**41.9**	14.9	6.6	0.79	0.84
pLDDT 1M	–	–	3.27	**41.8**	15.4	6.3	0.85	0.83
	+	1.0	3.28	40.7	14.3	6.5	0.85	
	+	3.0	3.25	41.7	17.2	6.5	0.84	
	+	10.0	3.24	41.6	16.1	6.6	0.85	
LDDT chain 42K	–	–	3.69	40.8	15.1	6.7	0.77	
	+	1.0	3.57	40.9	15.7	6.6	0.85	
	+	3.0	3.63	**41.7**	15.2	6.9	0.84	0.85
	+	10.0	3.59	41.6	15.2	6.8	0.83	
pLDDT chain 1M	–	–	3.29	40.5	16.1	6.6	0.81	
	+	1.0	3.36	40.8	17.1	6.5	0.88	
	+	3.0	3.30	41.0	15.0	6.5	0.85	
	+	10.0	3.31	**41.8**	15.4	6.3	0.87	0.85
pLDDT balanced 1M	–	–	2.63	**42.1**	15.8	6.4	0.75	0.83
pLDDT balanced 10M	–	–	2.43	42.0	14.9	7.0	0.76	
pLDDT balanced 60M	–	–	2.40	42.1	16.5	6.3	0.73	
pLDDT chain balanced 1M	–	–	2.45	41.1	18.0	6.1	0.75	
pLDDT chain balanced 10M	–	–	2.24	41.3	17.0	6.7	0.74	
pLDDT chain balanced 60M	–	–	2.21	**41.9**	17.5	6.3	0.73	0.83

Table 11: Evaluation results of Protein Infilling task, trained without (top row) and with SC regularization (all other rows). The table combines all the choices for AFDistill pretraining and showing their validation accuracy, as well as the corresponding performance on the downstream application. We select the best performance for each experiment based on the highest recovery rate (marked in bold).

REFERENCES

[1] Tsung-Yi Lin, Priya Goyal, Ross Girshick, Kaiming He, and Piotr Dollár. Focal loss for dense object detection. In *Proceedings of the IEEE international conference on computer vision*, pages 2980–2988, 2017.

[2] Jacob Devlin, Ming-Wei Chang, Kenton Lee, and Kristina Toutanova. BERT: Pre-training of deep bidirectional transformers for language understanding. *arXiv preprint arXiv:1810.04805*, 2018.

[3] Bowen Jing, Stephan Eismann, Patricia Suriana, Raphael JL Townshend, and Ron Dror. Learning from protein structure with geometric vector perceptrons. *arXiv preprint arXiv:2009.01411*, 2020.

[4] Chloe Hsu, Robert Verkuil, Jason Liu, Zeming Lin, Brian Hie, Tom Sercu, Adam Lerer, and Alexander Rives. Learning inverse folding from millions of predicted structures. *bioRxiv*, 2022.

[5] Zhanghao Wu, Paras Jain, Matthew Wright, Azalia Mirhoseini, Joseph E Gonzalez, and Ion Stoica. Representing long-range context for graph neural networks with global attention. *Advances in Neural Information Processing Systems*, 34:13266–13279, 2021.

[6] Wengong Jin, Jeremy Wohlwend, Regina Barzilay, and Tommi Jaakkola. Iterative refinement graph neural network for antibody sequence-structure co-design. *arXiv preprint arXiv:2110.04624*, 2021.

[7] Ahmed Elnaggar, Michael Heinzinger, Christian Dallago, Ghalia Rihawi, Yu Wang, Llion Jones, Tom Gibbs, Tamas Feher, Christoph Angerer, Martin Steinegger, et al. ProtTrans: towards cracking the language of life's code through self-supervised deep learning and high performance computing. *arXiv preprint arXiv:2007.06225*, 2020.
